# Reverse Vaccinology: An Approach for Identifying Leptospiral Vaccine Candidates

**DOI:** 10.3390/ijms18010158

**Published:** 2017-01-14

**Authors:** Odir A. Dellagostin, André A. Grassmann, Caroline Rizzi, Rodrigo A. Schuch, Sérgio Jorge, Thais L. Oliveira, Alan J. A. McBride, Daiane D. Hartwig

**Affiliations:** 1 Núcleo de Biotecnologia, Centro de Desenvolvimento Tecnológico, Universidade Federal de Pelotas, Pelotas RS 96100-000, Brazil; grassmann.aa@gmail.com (A.A.G.); ccrizzi@yahoo.com.br (C.R.); schuch.biotec@gmail.com (R.A.S.); sergiojorgevet@hotmail.com (S.J.); thais.larreoliveira@gmail.com (T.L.O.); alan.mcbride@ufpel.edu.br (A.J.A.M.); 2 Departamento de Microbiologia e Parasitologia, Instituto de Biologia, Universidade Federal de Pelotas, Pelotas RS 96100-000, Brazil; daianehartwig@gmail.com

**Keywords:** reverse vaccinology, leptospirosis, *Leptospira*, genomics, vaccine antigen, bioinformatics, genome mining

## Abstract

Leptospirosis is a major public health problem with an incidence of over one million human cases each year. It is a globally distributed, zoonotic disease and is associated with significant economic losses in farm animals. Leptospirosis is caused by pathogenic *Leptospira* spp. that can infect a wide range of domestic and wild animals. Given the inability to control the cycle of transmission among animals and humans, there is an urgent demand for a new vaccine. Inactivated whole-cell vaccines (bacterins) are routinely used in livestock and domestic animals, however, protection is serovar-restricted and short-term only. To overcome these limitations, efforts have focused on the development of recombinant vaccines, with partial success. Reverse vaccinology (RV) has been successfully applied to many infectious diseases. A growing number of leptospiral genome sequences are now available in public databases, providing an opportunity to search for prospective vaccine antigens using RV. Several promising leptospiral antigens were identified using this approach, although only a few have been characterized and evaluated in animal models. In this review, we summarize the use of RV for leptospirosis and discuss the need for potential improvements for the successful development of a new vaccine towards reducing the burden of human and animal leptospirosis.

## 1. Introduction

The worldwide incidence of leptospirosis is increasing year on year, from an initial estimate of 500,000 cases in 1999 [1] to over one million cases and 60,000 fatalities in 2015 [2]. Leptospirosis is a major public health problem in developing, impoverished countries and is responsible for economic losses in animal production [3], although the true impact is unknown. Current vaccines have not changed much in the last 100 years; they are based on inactivated whole-cell preparations (bacterins) or cell membrane extracts that are associated with several side-effects; the vast majority are for animal use only, although a handful of countries have licensed bacterins for human use [4]. In the last two decades, research has focused on the classical identification of antigens for the development of recombinant vaccines against leptospirosis, reviewed in [5]. While some progress has been made, the development of a broad-range vaccine remains elusive. However, during this time, the vaccine candidate discovery process underwent several major advances following the so-called “-omics boom”. The ever-increasing numbers and refinements in the quality of pathogenic genome sequences and bioinformatics tools, combined with advances in pathogenesis and immunology, led to the development and establishment of the process known as reverse vaccinology (RV) [6]. Rather than select individual proteins for evaluation, the RV approach analyses the entire genome of the pathogen and uses bioinformatics to reduce the number of potential targets, to approximately 500 proteins, followed by their high-throughput production as recombinant proteins and subsequent evaluation as potential vaccine candidates in the laboratory. While RV has been applied to many infectious diseases [7,8,9,10,11,12], there are few reports of its use in the field of leptospirosis. In this review, we highlight the importance of leptospirosis and the urgent need for the development of new recombinant vaccines, and we focus on the application of RV to leptospirosis.

## 2. *Leptospira* and Leptospirosis

Leptospirosis is a zoonotic disease caused by pathogenic spirochetes belonging to the genus *Leptospira* [13]. To date, 22 species of *Leptospira* have been identified, including 15 infectious (includes pathogenic and intermediately pathogenic *Leptospira* spp.) and seven non-infectious (saprophytic) species [14,15]. In addition to taxonomic classification, leptospires are traditionally classified serologically into serogroups and serovars. The antigenic complexity and diversity of leptospiral antigens, particularly lipopolysaccharide (LPS), have resulted in the identification of over 300 serovars [14]. Antigenically-related serovars are grouped into 24 serogroups for convenience. This genus is not classified by Gram staining because the thin bacterial cell is difficult to see by light microscopy. However, *Leptospira* spp. are diderms; they are structurally similar to Gram-negative bacteria, with an inner (IM) and an outer membrane (OM) and a peptidoglycan layer in between [16]. A distinctive leptospiral feature is the presence of two periplasmic flagella that are responsible for their characteristic motility that is observed in vitro and in vivo. The OM is spanned by transmembrane β-barrel proteins (OMP), mainly involved in the transport of molecules [17]. Lipoprotein coding DNA sequences (CDS) are abundant in the genome, and many lipoproteins have been described attached to the OM and exposed on the bacterial surface. *Leptospira* spp. have two chromosomes, and the majority of the genes code for hypothetical, uncharacterized proteins with no known orthologues [18]. Although very few tools are available for the genetic manipulation of *Leptospira* spp., a large number of mutants have been generated that have improved our understanding of pathogenesis [19].

Pathogenic *Leptospira* spp. are maintained in the environment by asymptomatic hosts that carry them in their kidneys and are subsequently shed in their urine. Rodents are the most frequent source of *Leptospira* spp. in urban environments, while domestic, farm and wild animals are important sources of transmission in rural areas [3]. In addition, leptospires are the only spirochetes that can survive outside of a host. Susceptible hosts can be infected by direct contact with the bodily fluids of infected animals or indirectly by exposure to leptospires in a contaminated environment. Transmission is usually related to wet environments, such as puddles, open sewers and flooding, where leptospires can survive for months outside the host [20]. Humans are considered accidental hosts; there are few reports of person to person transmission [21], such that it is considered irrelevant. Once the skin or mucosal barrier has been crossed in a susceptible host, leptospires spread rapidly through the blood stream to all organs and tissues during the acute phase of the disease. The host innate and adaptive immune system remove leptospires from the bloodstream; however, leptospires can colonize immune-privileged sites, such as the kidney [13]. The human disease can manifest as a mild, self-limiting febrile illness or as a severe, icteric disease with a fatal outcome. Due to the undifferentiated symptoms, leptospirosis is commonly misdiagnosed as other acute febrile syndromes. Symptoms of severe leptospirosis can include renal and hepatic dysfunction, uveitis, meningitis and hemorrhage. Leptospirosis pulmonary hemorrhage syndrome (LPHS) was reported as a clinical complication with a mortality rate of over 50% [22,23]. The host immunological mechanisms in response to leptospirosis are not completely understood. However, the immune response is predominantly biased towards the production of antibodies against leptospiral LPS. Pathogenic *Leptospira* spp. are resistant to complement-mediated killing; consequently, leptospiral clearance from tissues is probably mediated by opsonophagocytosis [24]. Antibiotic treatment is recommended, especially if started shortly after the onset of symptoms [25].

The diagnosis of leptospirosis is predominantly performed by serological tests. The microscopic agglutination test (MAT) is recommended by the World Health Organization (WHO), together with culture isolation [26]. The MAT is a highly subjective diagnostic test and requires paired serum samples collected at least two weeks apart, rendering it impractical for the clinical diagnosis of the disease and patient management. Underreporting and the frequent misdiagnosis of leptospirosis are responsible for the difficulty in accurately determining the real impact of the disease [4]. A recent report estimated the global burden of leptospirosis to be 1.03 million cases and 58,900 deaths worldwide yearly [2,27]. The impact of leptospirosis is higher in the developing world due to poor infrastructure, healthcare systems and the tropical climate. An effective vaccine is urgently required to prevent leptospirosis.

## 3. Conventional Vaccines Available against Leptospirosis

The first report of a vaccine for leptospirosis prophylaxis was published in 1916 [28], composed of whole inactivated *Leptospira* cells (bacterin), reviewed in [29]. Several physical (heat, irradiation, freeze-thawing) and chemical (formalin, phenol, ethanol) methods have been used to inactivate leptospires; however, bacterin preparations include contaminating medium components resulting in serious side-effects. In addition, the immune response induced by a bacterin is based on a humoral response to leptospiral LPS. As LPS is a thymus-independent antigen and does not induce the production of memory cells, bacterins induce short-term immunity, and there is a need for annual boosts. Due to the variable immunogenicity of LPS, bacterin vaccines are serovar-specific, protecting against the serovars included in the preparation with little or no heterologous protection. Therefore, the use of bacterin in humans has not been wide, and their use is permitted in only a few countries (e.g., China, Cuba, Japan and France), reviewed in [25].

The vaccination of cattle, pigs and dogs is common worldwide, and bacterin vaccines include locally-isolated serovars where possible [3]. This approach requires ongoing surveillance, initially to identify the predominant serovars and then to detect the appearance of any new serovars [30]. Canine bacterin vaccines tend to be based on four serovars and are used in many countries. In Brazil, these vaccines are produced locally or are imported; they contain serogroups Canicola and Icterohaemorrhagiae and frequently include serovars Pomona and Grippotyphosa [31]. Commercial cattle vaccines may contain up to eight serovars, including Hardjo, Icterohaemorrhagiae, Canicola, Grippotyphosa and Wolfii [3]. However, there is some controversy regarding the use of these vaccines and their ability to protect against renal colonization, bacterial shedding, reproductive failure and even infection in cattle [3,32].

## 4. Recombinant Vaccines Based on Classically-Identified Antigens

Recombinant vaccines represent an attractive strategy to overcome the limitations of conventional vaccines and successfully prevent leptospirosis, especially when considering safety and cross-protection [5]. Several experimental recombinant vaccines against leptospirosis have been evaluated to date, with variable efficacy. The first recombinant subunit vaccine evaluated was based on the porin OmpL1 and the lipoprotein LipL41 [33]. These proteins were originally discovered using non-ionic detergent solubilization, phase partitioning, immunoprecipitation and freeze-fracture electron microscopy [34,35]. In combination, recombinant OmpL1 and LipL41 induced homologous protection against challenge with *L. kirschneri* serovar Grippotyphosa. However, protection was observed only in one out of three experiments. While several groups have re-evaluated these proteins [36,37,38], it is not clear if they can induce a cross-protective or sterile immunity against leptospirosis.

The leptospiral lipoprotein with a molecular mass of 32 kDa, LipL32, is unquestionably the most studied leptospiral protein. LipL32 was localized to the OM by detergent fractioning [39], and this was followed by a flood of information; LipL32 is: present and highly conserved in all pathogenic *Leptospira* spp. [40], the most abundant leptospiral protein [41], expressed during infection [39] and in infection-mimicking simulations [42]. In addition, LipL32 binds extracellular matrix components [43,44] and was described as a surface-exposed protein [45]. However, the location of LipL32 in the OM of *Leptospira* spp. was recently re-evaluated and was reported to have a subcellular location, on the inner leaflet of the OM [46]. Furthermore, a *lipL32* gene knockout did not impair leptospiral virulence [47]. LipL32 was thoroughly evaluated as a recombinant subunit vaccine [48], DNA vaccines [49], *Mycobacterium bovis* strain BCG and adenovirus-vectored vaccine [50,51] and several adjuvants [52,53]; however, when a rigorous statistical analysis is performed on the data, the results were disappointing [5,29]. The failure of LipL32 to protect against leptospirosis may be explained by the possible subcellular location of LipL32.

The most promising results regarding protection against leptospirosis were achieved using the leptospiral immunoglobulin-like (Lig) proteins. LigA and LigB have a highly conserved N-terminal region and a less conserved C-terminal region. Both regions are immunogenic during infection, allowing their original identification by screening of *L. interrogans* and *L. kirschneri* expression libraries with convalescent human sera [54]. While there are reports that LigB can induce protection in animal models [55,56], LigA (the N-terminal region) induced an unequivocal immune protection in the hamster model using recombinant protein immunization [50,57]. However, the *ligA* gene is present only in three *Leptospira* spp. (*L. interrogans*, *L. kirschneri* and *L. alstonii*), making it difficult for a LigA vaccine to broadly protect against leptospirosis.

**Figure 1:**
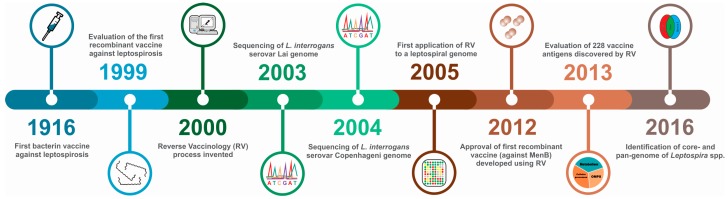
Timeline of vaccine and reverse vaccinology events for leptospirosis. 1916, the first classic bacterin vaccine against leptospirosis was produced and evaluated in a guinea pig model [28]; 1999, first evaluation of a recombinant subunit vaccine, based on OmpL1 and LipL41, tested in a hamster model [33]; 2000, Rino Rappuoli coined the term “reverse vaccinology” (RV) for the discovery and laboratory evaluation of vaccine candidates based on an analysis of the entire genome [6]; 2003 and 2004, publication of the first genome sequences of *L. interrogans* serovars Lai and Copenhageni, respectively [60,61]; 2005, first application of RV to the *L. interrogans* serovar Copenhageni genome [62]; 2012, the first vaccine discovered using RV was licensed in Europe [63]; 2013, first application of the complete RV process; 238 potential vaccine candidates from the *L. borgpetersenii* serovar Hardjo genome were evaluated in the hamster model of leptospirosis [64]; 2016, two independent groups published the first reports of the core- and pan-genome of *Leptospira* spp. [65,66].

Several other OMPs and OM lipoproteins were identified using classical methods (i.e., non-RV), reviewed in [17,29,58], and some were also evaluated as vaccine antigens, such as LipL21 [59]. However, these approaches tended to identify abundant proteins, such as LipL32, LipL21, LipL36 and FlaB, and were biased towards the identification of proteins that were not necessarily prospective vaccine candidates, but that were easily isolated from leptospiral cell. As discussed next, the use of RV proposes to improve the identification and evaluation of leptospiral vaccine candidates. A timeline of the major discoveries is presented in Figure 1.

## 5. The Reverse Vaccinology Process

**Figure 2:**
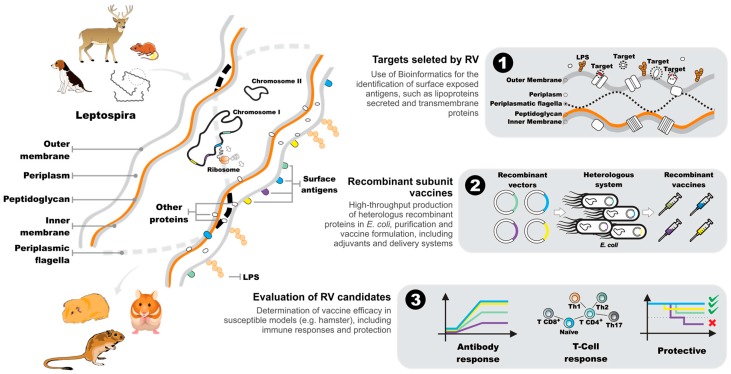
Schematic diagram of the reverse vaccinology process. The structure of the leptospiral cell and associated proteins is shown. Overview of the RV process: (1) selection of proteins from the genome sequence; (2) production of the recombinant subunit vaccines; and (3) evaluation of the RV candidates: protein-related humoral and cellular responses and protection against lethal challenge. LPS, lipopolysaccharide.

The advent of high-throughput sequencing technology and the subsequent cost reduction associated with DNA sequencing has led to a significant increase in the number of genome sequencing projects, in both public and private sectors. This has led to a demand for new bioinformatics tools to keep up with the ever-increasing amounts of sequence data being generated. The availability of these new bioinformatics tools, together with the failure to develop a vaccine against meningococcus B during the 1990s, led Rino Rappuoli and colleagues to propose a process for the identification of vaccine candidates directly from the genome sequence [6]. They coined the term “reverse vaccinology” to describe this novel approach that starts with the genome rather than a laboratory grown pathogen. As it is not feasible to evaluate all of the thousands of proteins encoded in a genome, a process was developed to screen for proteins that were likely to be exposed on the surface of the target microorganism. It was based on the prediction of protein structural features, such as: integral transmembrane β-barrel arrangements; transmembrane alpha-helices; signal peptides; lipoprotein lipoboxes; and secreted proteins. The original bioinformatics software and recent advances applicable to RV have been reviewed extensively elsewhere; see, e.g., [67,68]. This provided a rigorous analysis and the identification of candidate antigens for subsequent evaluation in the laboratory. The technique was originally proposed almost 20 years ago, and the first vaccine discovered using the RV process was licensed in Europe in 2012. Appropriately, it was the 4CMenB vaccine for the control of meningococcal B disease, now marketed worldwide as Bexsero by GlaxoSmithKline [63]. The reverse vaccinology process (RV) has been applied to a diverse range of infectious diseases including: malaria, tuberculosis, leishmaniosis and leptospirosis. However, the success of the approach requires, in addition to a genome sequence, a high-throughput cloning strategy, an animal model and in vitro screening assays to rapidly evaluate the potentially hundreds of vaccine candidates identified during the bioinformatics step. The RV process is summarized as applied to *Leptospira* spp.; see Figure 2.

## 6. Leptospiral Genomes as a Source of Vaccine-Related Information

The first two sequenced and published leptospiral genomes were released in 2003 and 2004, for *L. interrogans* serogroup Icterohaemorrhagiae serovars Lai [61] and Copenhageni [60], respectively. These were followed by the genome sequences for the saprophyte *L. biflexa* [69], the intermediate pathogen *L. licerasiae* [70] and the pathogens *L. borgpetersenii* [71] and *L. santarosai* [72]. In 2011, an international collaboration, the Leptospira Genome Project, was established with the objective to sequence the genomes of all known *Leptospira* spp. In addition, several individual research groups have contributed by sequencing the genomes of newly-isolated strains [15,30,73,74]. To date, hundreds of leptospiral genome sequences are available in public databases, e.g., more than 280 *L. interrogans* genome sequences are accessible. Most of the genome sequences available are draft genomes, with only a few dozen high-quality or finished genome sequences [66].

The genome of *Leptospira* spp. ranges in size from 3.8–4.7 MB and is distributed between two chromosomes [65,66,71]. The smallest genome encodes 2770 CDS (*L. borgpetersenii* strain JB197) [71] and the largest 4582 CDS (*L. alexanderi* strain L 60) [66], and the average GC content is low, ranging from 35%–45%. Perhaps one of the most distinguishing features of *Leptospira* genomes is the number of proteins with no orthologues in other genera. Picardeau and colleagues compared the genome of the saprophytic *L. biflexa* to that of the pathogen *L. interrogans* and found that 78% of the pathogen-unique proteins had no assigned function. The use of the term unknown in the annotation of recently published genomes has reduced due to improvements in the algorithms used to assign function. However, a large number of leptospiral proteins with potentially important roles in pathogenicity and host-adaptation have not been studied or characterized. Plasmids and mobile genetic elements were described in saprophytic *Leptospira* spp. over 15 years ago, allowing relatively easy genetic manipulation of *L. biflexa* [75,76,77]; while the discovery of plasmids in pathogenic species was a recent event [78]. The limited tools for genetic manipulation of pathogenic *Leptospira* spp. has impaired the discovery of protein function and essential virulence factors. Nevertheless, new technologies, such as the CRISPR/Cas system, promise advances in the genetic manipulation of this bacteria, reviewed in [66].

The comparative analysis of genome sequences from different *Leptospira* spp. has provided insights into the mechanisms involved in pathogenesis [60,69]. Recently, Fouts and co-workers sequenced and analyzed the genomes of 20 species of *Leptospira*, the core-genome included 1764 genes, and they identified 17,477 genes in the pan-genome [66]. When paralogs were counted as one gene, the sizes of the core- and pan-genomes were reduced to 1592 and 13,822 genes, respectively. In parallel, Xu and colleagues analyzed the genomes of 18 *Leptospira* spp. and identified 1023 genes in the core-genome and 57,765 genes in the pan-genome [65]. In both reports, the pan-genome was considered open, containing many more novel genes than the core-genome, including strain-specific genes. The core-genome of the pathogenic *Leptospira* spp. contains important information on potential vaccine candidates, including virulence factors central to the development of recombinant vaccines. Xu and colleagues (2016) reported that approximately 20% of the core-genome encoded toxins and other virulence genes [65]. The function of these conserved virulence factors and their potential as vaccine candidates needs to be determined in the laboratory.

Although RV has not been extensively applied to leptospiral genomes, to date, there are 10 reports in the literature that have used in silico genome mining towards the identification of leptospiral vaccine candidates. The RV process has been applied either completely [62,64] or just using the in silico analysis to select a small subgroup (<20) of proteins [38,79,80,81,82,83,84,85]; see Table 1. The first report was by a group of researchers at the Butantan Institute in Brazil; they reported that approximately 20% of CDS in the *L. interrogans* serovar Copenhageni genome contained a signal peptide, transmembrane domains, lipoprotein sequence motifs or were orthologues of OMPs. The list was further refined to include only hypothetical, unknown proteins with either a signal peptide or a lipoprotein motif. The final list included 206 CDS, 175 of which were successfully cloned and expressed as recombinant proteins in *Escherichia coli* [62]. The proteins were evaluated by Western blotting (WB) using sera from leptospirosis patients; 16 recombinant proteins were recognized and were predicted to include a signal peptide. The next RV report compared the Lai and Copenhageni genomes: 3672 orthologues were identified; they excluded 605 proteins that had orthologues in the human genome, resulting in 616 orthologues predicted to encode surface-exposed proteins [85]. In a novel approach, comparative genome hybridization (CGH) studies were used to reveal that 565 of the CDS located to the OM were conserved among 10 pathogenic serovars. Furthermore, an analysis of the transcriptome found that 1427 genes were significantly upregulated when cultured in vitro at 37 °C. The intersect between the set of potentially surface-exposed proteins and that of the transcriptome identified a group of 226 CDS. Neither of the last two studies evaluated the identified proteins in protection studies.

The next five studies applied the RV process to one or more genomes [80,81,84,86] or was the continuation of a previous RV project [82]. While 12 or fewer proteins were evaluated in each study, these were the first reports that included protection studies using vaccine candidates identified by RV. Chang and co-workers identified 10 putative OMPs that had no orthologues in GenBank and two OMPs with orthologues in *M. tuberculosis* [84]. While not strictly an RV study, the OMPs were identified from the LeptoList database of the *L. interrogans* serovar Lai annotated genome [61]. Of the 12 OMPs, three demonstrated efficacies that ranged from 51%–100%; however, none induced significant protection (*p* > 0.05, Fisher’s exact test) in the hamster model due to a high number of survivors (43%–50%) in the control groups. Atzingen and colleagues published an update of the RV project from 2005 [62]; they evaluated an additional three OMPs as vaccine candidates in the hamster model of leptospirosis [82]. The recombinant proteins induced fairly robust antibody responses in immunized hamsters; however, vaccine efficacy ranged from 13%–38% and was not significant (*p* > 0.05, Fisher’s exact test). In another report, bioinformatics was used to identify six putative OMPs containing OmpA-like domains in the *L. interrogans* serovar Pomona genome, although there are no details as to how this was achieved [81]. Although one of the recombinant proteins protected 67%–80% of vaccinated hamsters, it was not significant (*p* > 0.05, Fisher’s exact test) due to survivors in the control groups. The only reports based on RV, and that demonstrated significant protection, identified lipoproteins in the *L. interrogans* serovar Copenhageni strain Fiocruz L1-130 genome [80,86]. The authors used bioinformatics to identify proteins containing transmembrane helices, signal peptides, lipoboxes and their predicted cellular location. The initial screen identified 211 proteins predicted to contain a signal peptide and a transmembrane domain; an additional 15 proteins orthologous to vaccine candidates identified by RV in other pathogens were also included. Eight of the most promising targets were cloned and expressed as recombinant proteins in *E. coli*. The initial study evaluated the immunogenicity of the recombinant proteins in mice and similarity to the native protein by Western blot using convalescent patient sera [80]. In a follow-up study, one of the proteins, LemA, protected 88% of hamsters when a prime-boost vaccination strategy was used [86]. Although this was only a pilot study, protection was significant (*p* < 0.005, Fisher’s exact test) and even though sub-lethal infection was observed among surviving animals, LemA is a potential vaccine candidate that merits further investigation.

The following reports used several in silico approaches to identify potential vaccine candidates; however, none of the targets was evaluated in the laboratory. An analysis of 74 OMPs that are conserved among four genomes of *L. interrogans* and *L. borgpetersenii* reported the presence of immunogenic T-cell epitopes [79]. The most prominent antigen, the cation efflux system membrane protein (CzcA), contained four immunogenic epitopes. In the most extensive computational approach to identify the subcellular location of leptospiral proteins published to date, 114 putative extracellular and 63 putative OM proteins were identified [83]. Although not directly focused on the identification of vaccine targets, the putative transporter proteins identified in the *L. interrogans* and *L. borgpetersenii* genomes represent potential vaccine candidates, especially those located in the OM [89]. It would be interesting to reanalyze the target proteins using a more rigorous RV process to confirm their localization.

The most complete application of RV to leptospirosis identified 262 proteins in the *L. borgpetersenii* serovar Hardjo strain L550 genome that were predicted to be either secreted, located in the OM or to be lipoproteins [64]. Of these, a total of 238 recombinant proteins (223 unique proteins) were successfully produced in *E. coli*. Pools of proteins were evaluated in a hamster colonization model, and the endpoint was prevention of infection. None of the 48 combinations of proteins prevented infection, even though the majority (70%) of the proteins were immunogenic. Interestingly, several proteins (e.g., LigB, LipL41, LipL32) that are known to induce at least a partially protective immune response were among those tested; however, they did not prevent renal colonization. The most critical step is the bioinformatics analysis, and given the extensive tools available, the analysis used was fairly simple. Only one bioinformatics algorithm (predictor) was used for the identification of the majority of the characteristics associated with secreted or surface-exposed proteins; the only exception was for lipoproteins (two predictors were used). The accurate identification of a particular type of OMP should be based on a consensus analysis of several predictors rather than a single method, reviewed in [90]. Thus, while the final RV analysis included LigB, LipL41 and LipL32, it is likely that non-surface-exposed proteins were erroneously identified and that potentially surface-exposed proteins were excluded due to the use of only one predictor. The kidney colonization model of leptospirosis is useful for the identification of potential vaccine candidates for animal vaccines. However, to date, sterile immunity was not induced by any of the subunit vaccine candidates evaluated in the lethal model of leptospirosis. Therefore, it would be interesting to evaluate these target antigens in a lethal model of leptospirosis.

## 7. Downstream Analysis of Candidate Reverse Vaccinology Targets

Bioinformatics is now ubiquitous in many fields that use genome-based information, and it has evolved to offer high levels of confidence in the prediction of potential vaccine candidates. However, further investigation in vitro of those antigens is still necessary and highly desirable before testing experimental vaccines in animal models. Advances in genomics, transcriptomics, proteomics and other fields have contributed to the rational selection of antigens and refinement of the vaccine candidates under evaluation. Comparative transcriptomics of *Leptospira* spp. grown in vitro and in a model that simulated infection identified antigens that underwent differential transcription in host-adapted leptospires [42]. Additional transcriptomics studies investigated the pattern of gene expression in leptospires exposed to temperature shift [91,92,93,94], increased osmolarity [95,96,97], iron starvation [98] and the presence of serum [99]. Only a limited number of transcripts were observed to be up- or down-regulated under different conditions; however, valuable information on a given pathogenic-related gene can be mined from them.

*Leptospira* proteomics has focused on global analysis [41,100,101,102], comparative analysis of the proteomes of different serovars [103,104,105], expression patterns related to environmental changes [100,106] and proteomics comparing in vitro and in vivo conditions [107,108]. An innovative mass-spectrometry-based strategy was employed to determine the absolute number of protein copies per *L. interrogans* cell [102]. In another global analysis of the *L. interrogans* proteome, 563 proteins were identified, 65 of them upregulated under in vivo-like conditions [100]. Such studies contribute to the rational selection of vaccine targets that are either upregulated and/or expressed at high levels during infection.

Recently, the post-translational modification (PTM) of leptospiral proteins has been suggested as an immune evasion strategy of leptospires and is possibly linked to the effectiveness of host immune responses [109,110]. Further studies are necessary to clarify the possible role of PTM on the protection induced by heterologous recombinant subunit vaccines. The RV process could be adapted to use an alternative heterologous expression system, e.g., *Pichia pastoris*, that is capable of PTM. The potential vaccine candidate LigA was expressed in *P. pastoris* and retained its efficacy [111]. The immunogenicity of leptospiral proteins during infection has been evaluated by one- or two-dimensional gel electrophoresis of whole-cell extracts and reaction with convalescent patient sera [45,62]. However, the immune response induced by vaccine preparations is usually evaluated by the quantification of specific antibodies against the vaccine antigen and quantification of mRNA levels of immune mediators (mostly cytokines) by qRT-PCR [112]. This limitation is a consequence of the use of hamsters as the standard animal model for acute leptospirosis and the lack of materials and reagents to better describe the cellular immune response in this model. The field of leptospirosis vaccine development would benefit either by additional biological tools to study the hamster immune system or by the improvement of leptospirosis models based on mice or rats, for which numerous reagents are commercially available. Consequently, there is no available in vitro assay that correlates the immune response induced by a vaccine preparation to the protection observed. RV studies on, e.g., *N. meningitides*, use a bactericidal antibody assay, a well-known correlate of protection, and this has contributed to the identification of vaccine candidates using RV [113]. Similarly, an immune correlate assay based on the opsonophagocytosis of *Staphylococcus aureus* was essential for the identification of vaccine antigens [114]. Recently, a correlation between IgG levels two weeks after immunization and the survival of hamsters immunized with a lipidated form of the LigA was suggested as a potential correlate of protection for leptospirosis [115]. However, this correlation was observed only for the mean values, and not all animals met this criterion. The development of a meaningful correlate of immunity for leptospirosis vaccines would not only reduce the number of animals used in experimentation, but potentially reduce the time to discovery of a new, protective vaccine.

## 8. Conclusions

A large amount of genomics and proteomics data is currently available, and several promising antigens have been identified using bioinformatics tools based on RV. The translation of this knowledge into effective vaccine candidates is fundamental and should be extensively explored. A major drawback in the application of the RV process to leptospirosis is the lack of immune correlates that can be used in laboratory assays. These assays are necessary in order to refine the list of potential vaccine targets that will be used to determine vaccine efficacy in an animal model. Given the breadth of bioinformatics tools that are available, the in silico analysis of leptospiral genomes could be significantly improved, increasing confidence that the predicted antigens are in fact surface-exposed proteins and therefore potential vaccines. As a large number of annotated proteins in *Leptospira* genomes does not have any known orthologues, RV represents the most promising approach for the discovery of a recombinant vaccine, thereby reducing the burden of leptospirosis.

## Figures and Tables

**Table 1:** Reverse vaccinology projects applied to *Leptospira* spp.

Serovars ^a^	No. CDS	RV Targets	Targets Screened	Localization	Expt. Data	% Efficacy ^d^	Fisher (*p*)	Reference
Cop	3737	206	16	OMP/LIP	WB	ND	ND	[62]
Lai-1/Cop	3672 ^b^	226	NA	OMP/IM/PS/SEC	CGH/MA	ND	ND	[85]
Lai-1/Pom	4727/3741	NK	12	OMP	RV/HML	51–100	>0.05	[84]
Lai-1/Cop	4727	177	ND	ND	ND	ND	ND	[83]
Cop	3737	206	3	OMP	WB/HML	12–38	>0.05	[82]
Pom	NK	6	6	OmpA-like	ELISA/CR/HML	43–80	>0.05	[81]
Cop	3530	226	8	LIP	ELISA/WB/HML	88	<0.01	[80,86] *
Lai-1/Cop/Har-1 & 2	2689 ^b^	74	12 ^e^ (9) ^c^	OMP	NA	ND	ND	[79]
Har-1	3412	262	238 (223) ^c^	OMP/LIP/SEC	ELISA/HKCM	0	ND	[64]
Cop/Lai-1 & 2/	3667/4727 & 3711/	63	12 (26) ^c^	SEC	NA	ND	ND	[87]
Har-1 & 2	3412 & 3277							

CDS, coding sequence; RV, reverse vaccinology; OMP, outer membrane protein; SE, surface-exposed; LIP, lipoprotein; PS, periplasmic space; IM, inner membrane; SEC, secreted; CGH, comparative genomic hybridization; MA, RNA microarray; WB, Western blot; CR, cytokine response; HML, hamster model of leptospirosis; HKCM, hamster kidney colonization model; NK, not known; ND, not determined; NA, not applicable. ^a^ Serovar/strain: Cop, *L. interrogans* serovar Copenhageni strain L1-130; Lai-1, *L. interrogans* serovar Lai strain 56601; Lai-2, *L. interrogans* serovar Lai strain IPAV; Pom, *L. interrogans* serovar Pomona; Har-1, *L. borgpetersenii* serovar Hardjo strain L550; Har-2, *L. borgpetersenii* serovar Hardjo strain JB197; ^b^ Conserved among indicated serovars; ^c^ Number of unique proteins; ^d^ Efficacy is expressed as the proportionate reduction in disease attack rate between the control and vaccinated groups [88]; ^e^ Peptides. * The RV analysis was published in 2011, and the protection study was published in 2013.
